# High-Dose Primaquine Induces Proximal Tubular Degeneration and Ventricular Cardiomyopathy Linked to Host Cells Mitochondrial Dysregulation

**DOI:** 10.3390/toxics11020146

**Published:** 2023-02-02

**Authors:** Atthasit Rabiablok, Borimas Hanboonkunupakarn, Khwanchanok Tuentam, Kamonpan Fongsodsri, Tapanee Kanjanapruthipong, Sumate Ampawong

**Affiliations:** 1 Department of Clinical Tropical Medicine, Faculty of Tropical Medicine, Mahidol University, 420/6 Ratchawithi Road, Ratchathewi, Bangkok 10400, Thailand; 2 Department of Tropical Pathology, Faculty of Tropical Medicine, Mahidol University, 420/6 Ratchawithi Road, Ratchathewi, Bangkok 10400, Thailand

**Keywords:** heart, kidney, liver, mitochondria, primaquine, toxicity

## Abstract

Primaquine (PQ) is the only antimalarial medication used to eradicate many species of *Plasmodium* gametocytes and prevent relapse in vivax and ovale malarias. PQ metabolites induce oxidative stress and impair parasitic mitochondria, leading to protozoal growth retardation and death. Collateral damage is also presented in mammalian host cells, particularly erythrocytes, resulting in hemolysis and tissue destruction. However, the underlying mechanisms of these complications, particularly the mitochondria-mediated cell death of the host, are poorly understood. In the present study, toxicopathological studies were conducted on a rat model to determine the effect of PQ on affected tissues and mitochondrial toxicity. The results indicated that the LD_50_ for PQ is 200 mg/kg. A high dose of PQ induced hemolytic anemia, elevated a hepatic enzyme (SGPT), and induced proximal tubular degeneration, ventricular cardiomyopathy, and mitochondrial dysregulation. In addition, PQ induced the upregulation of apoptosis-related proteins Drp-1 and caspase-3, with a positive correlation, as well as the pro-apoptotic mitochondrial gene expression of *Bax*, reflecting the toxic effect of high doses of PQ on cellular damage and mitochondrial apoptosis in terms of hepatotoxicity, nephrotoxicity, and cardiotoxicity. Regarding the risk/benefit ratio of drug administration, our research provides caution for the use of PQ in the treatment of malaria based on its toxicopathological effects.

## 1. Introduction

Malaria cases have decreased from 2000 to 2022 in the six countries of the Greater Mekong subregion, which includes Thailand, Viet Nam, Myanmar, Cambodia, China (Yunnan Province), and the Lao People’s Democratic Republic. However, recently more cases in the Western Pacific Region have significantly increased [1]. Malaria caused by *Plasmodium falciparum* and *P. vivax* continues to be a significant global infectious disease. Antimalarial drugs, such as chloroquine, sulfadoxine/pyrimethamine, mefloquine, quinine, artemisinin, artesunate, primaquine, and tafenoquine, each have their own therapeutic benefits and contraindications [2]. The risk/benefit ratio of drug administration should be carefully evaluated, particularly in relation to patient history and dosage. Primaquine (PQ), an 8-aminoquinolone, prevents relapses of vivax malaria and may kill gametocytes of falciparum malaria [3]. However, PQ has several side effects [3], including (i) abdominal discomfort at doses greater than 0.5 mg/kg, (ii) oxidative hemolysis, which is most vulnerable in patients with glucose-6-phosphate dehydrogenase (G6PD) deficiency, and is characterized by intravascular hemolysis, anemia, and hemoglobulinuric renal failure, and (iii) methemoglobinemia, especially in NADH-methemoglobin reductase deficient patients [4,5].

The precise mechanisms of action and toxicity of PQ are poorly understood. Most studies were conducted using clinical reports and experimental models involving rats, cell lines, fungi, and blood cells. Baker and colleagues reported that PQ exhibits dose-dependent selective toxicity for both stimulation and inhibition of host and parasite mitochondrial respiration [6]. In the liver, PQ is converted into multiple metabolites by cytochrome P450 and monoamine oxidase A [7]. These metabolites induce oxidative stress and impair parasitic mitochondria, resulting in growth retardation, most likely due to the damage of the labile Fe-S cluster and reactive oxygen species (ROS)-sensitive enzymes [8]. PQ metabolites can also destroy mammalian host cells, particularly erythrocytes. Unfortunately, studies on the toxic effects of PQ on the mitochondria of the host in terms of mitochondria-mediated cell death or apoptosis and other affected organs are still limited.

In the present study, lethal dose, 50% (LD_50_) of PQ and its associated toxicity were determined using a rat model. Based on toxicopathology studies, the affected organs and mitochondria in the liver and kidney were examined. Immunoelectron microscopy, immunohistopathology, clinicohematology, and quantitative reverse-transcription polymerase chain reaction (qRT-PCR) techniques were used to demonstrate histopathological alterations and mitochondrial architecture, homeostasis, energetic balance, and apoptosis in relation to PQ toxicity. As a safety criterion for the use of PQ in the treatment of malaria, the results reveal the role of PQ on mammalian tissue and mitochondrial toxicities involved with related pathogenesis.

## 2. Materials and Methods

### 2.1. Ethical Permission

This research protocol was approved by the Animal Care and Use Committee, Faculty of Tropical Medicine, Mahidol University, Bangkok, Thailand (Approval No. FTM-ACUC 006/2021). All animal experiments were conducted in accordance with Thailand’s Animal for Scientific Purposes Act, B.E. 2558 (A.D. 2015). Twenty-five healthy male Wistar rats (8 weeks old, 200–250 g) were procured from Nomura Siam International, Bangkok, Thailand. They were acclimated for 7 days after arrival and housed under strict hygienic conditions, including a 12/12 h dark/light cycle and temperature and humidity controls. They were fed ad libitum with reverse osmosis water and standard diet No. 082 (Perfect Companion Ltd., Bangkok, Thailand).

### 2.2. Experimental Procedure

In order to determine the LD_50_ of PQ and its toxicological effects on the liver and kidney mitochondria as well as other clinicopathological alterations, all rats were divided into five groups (five of each). Rats that were not treated were gavaged with sterile water, while treated rats received 100, 150, 200, or 250 mg/kg of PQ (The Government Pharmaceutical Organization, Thailand: Lot No. S640149) for 3 consecutive days. All rats were subjected to daily clinical observation, focusing on cardiovascular and respiratory vital signs, abnormal behavior, anxiety, and depression until 24 h after the last dose. The number of moribund, dead, and alive rats was recorded daily. Moribund rats were claimed as dead animals for the calculation of LD_50_. All surviving rats were terminated on day 7 by being humanely euthanized with isoflurane inhalation. An early endpoint was performed on the moribund rats before the termination date.

### 2.3. Sample Collection and Mitochondrial Extraction 

A full analysis was conducted on two experimental groups: (i) non-treated rats and (ii) rats that received the closest dose to the LD_50_ of PQ, as calculated by probit analysis. At the endpoint, exsanguination blood specimens were collected by cardiac puncture under isoflurane inhalation anesthesia, placed in an EDTA tube, and centrifuged at 1500× *g* for 15 min to separate plasma. The National Laboratory Animal Center of Mahidol University, Bangkok, Thailand, analyzed the complete blood count and blood clinical chemistry. The liver, kidney, heart, and spleen were removed and preserved in 10% neutral buffer formalin for histopathological and immunohistochemical examinations. The liver and kidney of terminated rats were immediately placed in ice-cooled mitochondrial extraction buffer (250 mM sucrose, 5 mM KH_2_PO_4_, 10 mM Tris-HCl, and 2 mg/mL BSA, pH 7.2) for mitochondrial extraction prior to electron microscopic and molecular analysis.

The liver and kidney mitochondria were extracted as described previously [9,10,11,12,13,14]. Each group’s liver and kidney were combined, homogenized in an extract buffer, and centrifuged at 1000× *g* and 4 °C for 5 min. The mitochondrial supernatant was gathered and centrifuged at 15,000× *g* and 4 °C for 2 min. The mitochondrial pellet was separated and washed multiple times with an extract buffer to eliminate other organelles and debris. Then, the pellets were separated into two portions. For electron microscopic examination, 200 µL of mitochondria were fixed in 2.5% glutaraldehyde in 0.1 M sucrose phosphate buffer (SPB) for 1 h, washed in 0.1 M SPB for 10 min three times, and stored in SPB at 4 °C. The remainder of the mitochondrial pellet was preserved in RNA later and stored at −20 °C for molecular study.

### 2.4. Electron Microscopic Study

To determine the mitochondrial apoptotic, dynamic, antioxidative, energetic, and homeostatic properties, which are determined by the expression of caspase-3, dynamin-related protein-1 (Drp-1), optic atrophy protein-1 (OPA-1), nuclear factor erythroid 2-related factor-2 (Nrf-2), haloacid dehalogenase-like hydrolase domain containing-3 (HDHD-3), and mitogen-activated protein kinase (MAPK), respectively, electron microscopic study was performed. Fixed mitochondria were washed with SPB and then refixed with osmium-tetroxide for 1 h. The samples were dehydrated in grading ethanol, infiltrated in grading LR White resin (EMS, USA), embedded in LR White resin, polymerized at 65 °C for 24–48 h, and sectioned to a thickness of 100 nm. Then, immunogold labeling assays were performed on every section. The sections were blocked with 50 mM glycine and 5% bovine serum albumin (BSA; EMS, USA) and incubated for 1 h at room temperature with caspase-3, Drp-1, OPA-1, Nrf-2, HDHD-3, and MAPK antibodies. The sections were incubated with immunoglobulin (Ig) G conjugated with 10 nm gold particles (EMS, USA) for 1 h. Silver enhancement was performed using the Aurion R-Gent SE-EM kit (EMS, USA). The sections were stained with lead citrate and uranyl acetate and evaluated using a transmission electron microscope (TEM; HT7700, HITACHI, Tokyo, Japan). In addition, mitochondrial architecture was examined, and the proportion of normal mitochondria/field was calculated.

### 2.5. Histopathological Study

In order to determine any toxicological effects of PQ on renal, hepatic, cardiac, and splenic tissues, a histopathological examination was conducted. Standard tissue processing was used to embed fixed specimens, which were then cut to a 5 µm thickness and stained with hematoxylin and eosin (H&E). Under a light microscope, histopathological alterations were evaluated and scored using the H-score (0–300), which was calculated by multiplying the severity score (0–3; 0—absent, 1—mild, 2—moderate, and 3—severe) by the extent of lesion distribution/tissue section (estimated ranges of 0–100%).

### 2.6. Immunohistochemical Study 

In order to evaluate PQ toxicity in relation to mitochondrial-induced apoptosis in the heart, immunohistochemical staining of Drp-1 and caspase-3 was performed. Prior to immunostaining, heart tissue sections were deparaffinized in xylene and rehydrated. Utilization of heat-induced antigen retrieval with citrate buffer, pH 6. After the sections were cooled, endogenous peroxidase was quenched with 3% *v*/*v* hydrogen peroxide in methanol. Sections were blocked with serum-free protein block (Dako, Glostrup, Denmark, X0909). Sections were incubated with antibodies against Drp-1, caspase-3, and labeled polymer HRP antimouse/rabbit EnVision kit (Dako, Glostrup, Denmark, K5007), respectively. The sections were stained with diaminobenzidine (DAB, DAKO, Glostrup, Denmark, K3468), counterstained with hematoxylin, and mounted permanently with DPX (Electron Microscopic Sciences, PA, USA).

Five random fields (at ×400) were examined for all rats in each group. Using a light microscope (BX51, Olympus^®^) and a digital camera (DP20, Olympus^®^), color images of the 640 × 480-pixel resolution were acquired for each specimen. Immunolabeling was then analyzed using semi-quantitative digitalized image analysis with ImageJ from the National Institutes of Health. Briefly, color images were converted to 8-bit images, the labeling area was located using the threshold mode, and the percentage area of expression was estimated using the number of black pixels in measuring mode. Thus, the H-score (0–300) was calculated by multiplying the intensity score (0–3; 0—absent, 1—mild, 2—moderate, and 3—severe) by the percentage expression area (0–100%).

### 2.7. qRT-PCR

qRT-PCR was performed using the iTaq Universal SYBR Green Supermix to identify mitochondrial apoptotic genes relevant to PQ-induced toxicity using pro-apoptotic (*Bax* and *Bnip3*) genes (Biorad, Hercules, CA, USA). In the CFX96 Touch™ Real-time PCR detector, the primer pairs were used, as shown in Table 1, for amplification and denaturation (Biorad, Düsseldorf, Germany) [15]. The individual gene expression levels were calculated using the 2^−∆∆Ct^ method. Next, *18S* expression levels f were used as a stable reference gene for accurate normalization of gene expression data.

### 2.8. Statistical Analysis

The data were statistically analyzed using IBM^®^ SPSS^®^ Statistic software version 20. Probit analysis was utilized to establish the LD_50_ of PQ. The percentage of intact mitochondria, percentage of positively labeled cells, H-score, and relative gene expressions were compared between groups using an independent *t*-test. Spearman’s correlation test was used to determine the correlation between measured parameters. The criterion for statistical significance was a *p*-value less than 0.05.

## 3. Results

### 3.1. Clinicohematological Data

Twenty-four hours after the last dose of PQ administered to any group, all rats in the 100 mg/kg and 150 mg/kg groups exhibited normal symptoms, whereas all rats in the 250 mg/kg group were dead. Three rats administered 200 mg/kg PQ exhibited abnormal clinical manifestations, including severe depression, ruffled fur, lateral recumbency, breathlessness, bradycardia, defecation and urination incontinence, and moribundity. The probit analysis revealed that the calculated LD_50_ of PQ was 198.386 mg/kg (95% confidence interval (CI): 74.417–217.715 mg/kg). In this study, rats that were not treated with PQ and rats that received 200 mg/kg of PQ were chosen for full-scale analysis.

Blood clinical chemistry and complete blood count (Table 2) revealed that white blood cell, neutrophil, blood urea nitrogen (BUN), and serum glutamate-pyruvate transaminase (SGPT) levels in 200 mg/kg PQ-treated rats were elevated compared to normal limits. Compared to normal ranges, the red blood cell (RBC), hemoglobin (HGB), hematocrit (HCT), mean corpuscular hemoglobin concentration (MCHC), and red cell distribution width (RDW) of rats treated with 200 mg/kg of PQ were reduced. However, a slightly high platelet (PLT) concentration was also detected in the non-treatment group compared to the norm. In addition, 200 mg/kg PQ-treated rats exhibited a significant increase in SGPT and creatinine compared to untreated rats, while RBC, HCT, MCHC, and RDW decreased significantly.

### 3.2. High Dose of PQ-Induced Renal Degeneration and Ventricular Cardiomyopathy 

**Figure 1:**
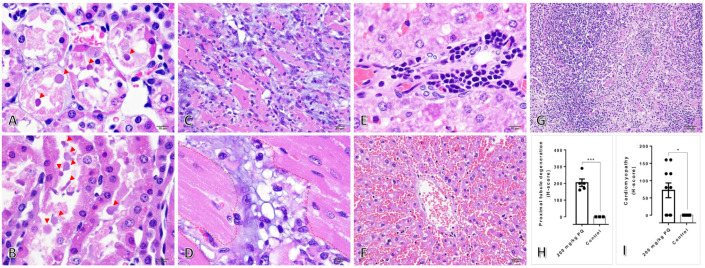
Histopathological changes in the kidney, heart, liver, and spleen in rat treated with 200 mg/kg of PQ: (**A**) Cross section of proximal convoluted tubules with extensive epithelial cell damage, pyknotic nuclei, and basophilic droplets deposition (arrow head). (**B**) Proximal convoluted tubules with longitudinal orientation exhibited vacuolated epithelial degeneration, nucleolar pyknosis, eosinophilic cytoplasm, and luminal basophilic droplets accumulation (arrow head). (**C**) Cardiomyopathy characterized by diffuse interstitial fibrosis, extensive accumulation of mononuclear cells, and slight neutrophil accumulation. (**D**) A higher magnification of (**C**) highlighting the area of the histopathological lesion (red dotted-line). (**E**) The presence of extramedullary hematopoiesis revealed erythroid cell infiltration in the periportal region of the liver. (**F**) Sinusoidal space congestion space with red blood cells in the liver. (**G**) Intact spleen showed normal red and white pulps area. (**H**,**I**) Histopathological scores, respectively, for proximal tubular degeneration and ventricular cardiomyopathy (H&E staining). For subsequent figures, statistical significance of comparisons are *, *p* < 0.05 and ***, *p* < 0.0001.

Evaluation of histopathological changes in rats treated with or without PQ was performed. The results revealed that 200 and 250 mg/kg of PQ caused proximal tubular degeneration and accumulation of basophilic droplets in the kidney, whereas different doses and the control group were unaffected. Additionally, ventricular cardiomyopathy was observed only in the hearts of rats administered 200 and 250 mg/kg of PQ. Figure 1 depicts histopathological appearances and their severity scores. The severity scores of these two histopathological findings were significantly greater in rats treated with 200 mg/kg PQ than in control rats. In addition, one rat in the 200 mg/kg PQ-treated group exhibited extramedullary hematopoiesis and sinusoidal congestion with red blood cells. However, rats treated with 200 and 250 mg/kg still had intact spleens.

### 3.3. High Dose of PQ Caused Mitochondrial Alteration in the Kidney and Liver 

**Figure 2:**
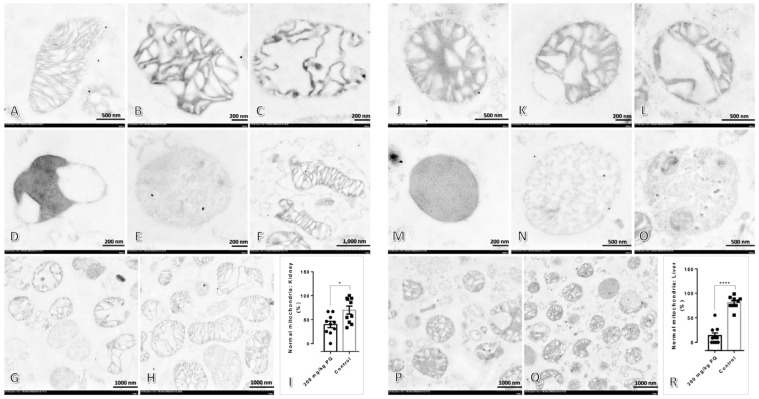
Electron micrographs of kidney and liver mitochondria in rats treated with or without 200 mg/kg of PQ: (**A**–**F**) Renal mitochondrial ultrastructure in six different forms: normal mitochondrion with double membrane and typical arrangement of cristae and matrix (**A**), swelling mitochondrion with an irregular pattern of cristae and moderate distension of matrix (**B**), spheroid mitochondrion with some losses of cristae and extensive distension of matrix (**C**), clumping mitochondrion with the loss of double membrane and cristae and the dense aggregation of matrix and its material (**D**), ghost mitochondrion with loss of all intact mitochondrial architecture and remaining with very fine granule appearance (**E**), and mitophagy—a giant mitochondrion ingested with few mitochondria (**F**). (**G**,**H**) Renal mitochondria architecture in rats with or without 200 mg/kg of PQ treatment, respectively. (**I**) The percentage of normal mitochondria in the non-treated kidney between treat and non-treat group. (**J**–**O**) Hepatic mitochondrial ultrastructure in six distinct forms, including intact, swelling, spheroid, clumping, ghost, and mitophagy. (**P**,**Q**) Hepatic mitochondrial architecture in rats treated with or without 200 mg/kg of PQ. (**R**) The proportion of normal liver mitochondria in the treated versus untreated group. The statistical significance of comparisons for subsequent figures is *, *p* < 0.05; and ****, *p* < 0.00001.

The mitochondrial architecture was examined by the ultrastructural changes described in our previous studies [9,12,13,14], as shown in Figure 2. The results demonstrated that rats treated with 200 mg/kg of PQ had significantly fewer normal mitochondria in their kidneys and livers than the control group.

### 3.4. Upregulation of Drp-1 and Caspase-3 Were Observed in the Kidney and Liver Mitochondria Induced by a High Dose of PQ 

**Figure 3:**
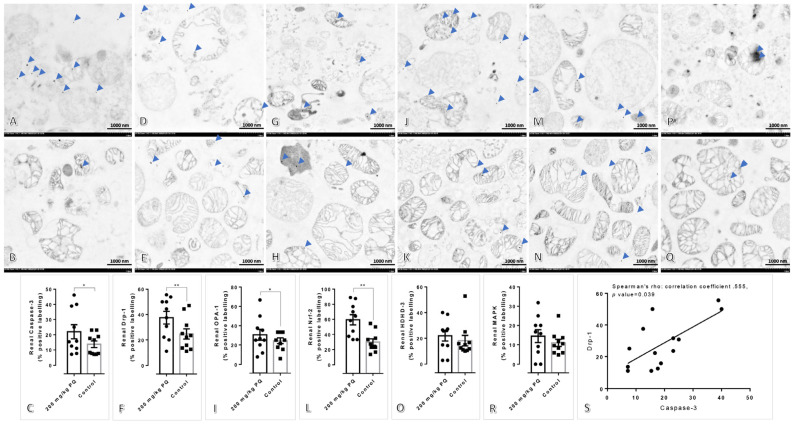
Immunogold labeling of caspase-3, Drp-1, OPA-1, Nrf-2, HDHD-3, and MAPK in renal mitochondria after treated with or without 200 mg/kg of PQ: (**A**,**D**,**G**,**J**,**M**,**P**) Renal mitochondrial architecture in rats treated with 200 mg/kg of PQ. (**B**,**E**,**H**,**K**,**N**,**O**) Renal mitochondrial architecture in non-treated rats. (**A**–**R**) Immunogold labeling of caspase-3, Drp-1, OPA-1, Nrf-2, HDHD-3, and MAPK in rats treated with or without 200 mg/kg of PQ, respectively. (**S**) Spearman’s correlation test between the levels of Drp-1 and caspase-3 in the renal mitochondria of rats treated with 200 mg/kg of PQ or not. The statistical significance of comparisons for subsequent figures is *; *p* < 0.05 and **; *p* < 0.01.

Immunogold labeling stained results showed that the expression of caspase-3, Drp-1, OPA-1, and Nrf-2 in renal mitochondria of rats treated with 200 mg/kg of PQ was significantly higher than that of control rats, whereas the expression of HDHD-3 and MAPK in renal mitochondria were comparable between the two groups (Figure 3). Concerning these expression levels, there was a significant positive correlation between Drp-1 and caspase-3 expression in the kidney.

**Figure 4:**
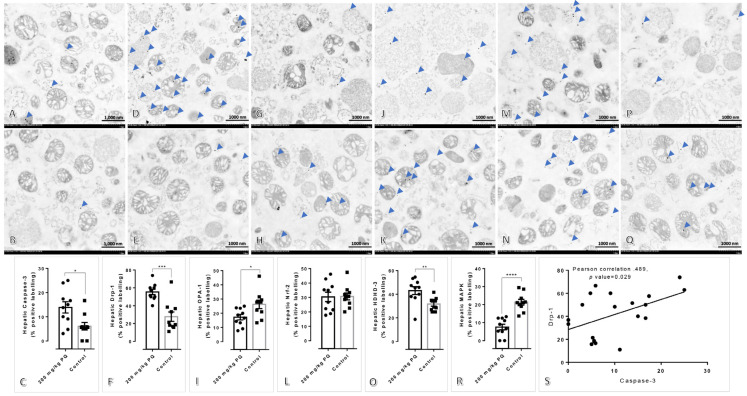
Immunogold labeling of caspase-3, Drp-1, OPA-1, Nrf-2, HDHD-3, and MAPK in hepatic mitochondria after treated with or without 200 mg/kg of PQ: (**A**,**D**,**G**,**J**,**M**,**P**) Hepatic mitochondrial architecture in rats treated with 200 mg/kg of PQ; (**B**,**E**,**H**,**K**,**N**,**O**) Hepatic mitochondrial architecture in non-treated rats; (**A**–**R**) Immunogold labeling of caspase-3, Drp-1, OPA-1, Nrf-2, HDHD-3, and MAPK in rats treated with or without 200 mg/kg of PQ, respectively. (**S**) Spearman’s correlation test between the level of Drp-1 and caspase-3 in the hepatic mitochondria of rats treated or non-treated with 200 mg/kg of PQ. The statistical significance of comparisons for subsequent figures is *, *p* < 0.05; **, *p* < 0.01; ***, *p* < 0.0001; and ****, *p* < 0.00001.

In addition, the expression of hepatic caspase-3, Drp-1, and HDHD-3 was significantly elevated in rats treated with 200 mg/kg of PQ compared to untreated rats (Figure 4). Hepatic OPA-1 and MAPK expressions were significantly lower in the treatment group than in the control group, whereas hepatic Nrf-2 expression was identical in both groups. There was a significant positive correlation between the expression of Drp-1 and caspase-3 in the liver, as observed in the kidney.

### 3.5. Upregulation of Drp-1 and Caspase-3 were Observed in the Heart Induced by a High Dose of PQ 

**Figure 5:**
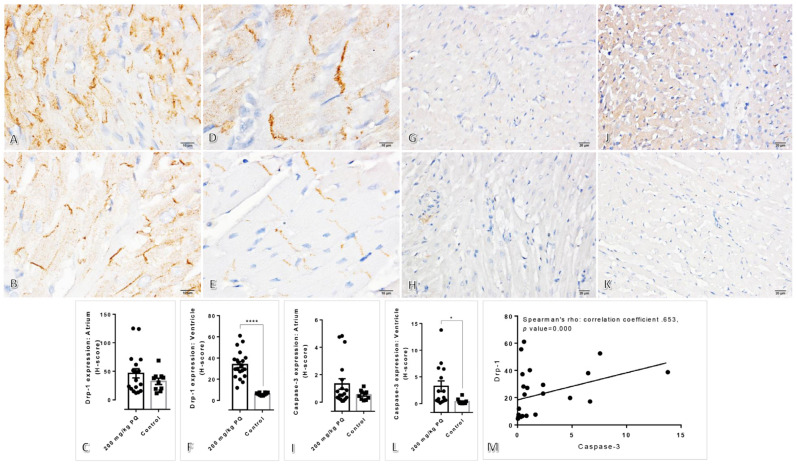
Immunohistochemical staining for cardiac Drp-1 and caspase-3 in rat-treated with or without 200 mg/kg of PQ: (**A**–**F**) Atrium and ventricle immunolabeling of Drp-1 in rats treated with (**A**,**D**) or without (**B**,**E**) 200 mg/kg of PQ. (**G**–**L**) Atrium and ventricle immunolabeling of caspase-3 in rats treated with (**A**,**D**) or without (**B**,**E**) 200 mg/kg of PQ. (**M**) The Spearman’s correlation test between the level of Drp-1 and caspase-3 in the cardiac mitochondria both rats treated with or without 200 mg/kg of PQ. For subsequent figures, statistical significance of comparisons is *, *p* < 0.05; and ****, *p* < 0.00001.

Immunohistochemical staining of Drp-1 and caspase-3 in the heart was also performed due to the unanticipated discovery of ventricular cardiomyopathy in rats treated with 200 mg/kg of PQ, although the initial objective of this study was not to isolate heart mitochondria. The immunochemical outcomes, however, were consistent with immunogold staining. Next, the atrial and ventricular regions were analyzed. Compared to untreated rats, the expression of Drp-1 and caspase-3 on the ventricle of rats treated with 200 mg/kg PQ was significantly upregulated, as shown by the results (Figure 5). Unlike the ventricle, both markers were expressed identically in the atrium. Correspondingly, a positive correlation was found between the expression of Drp-1 and caspase-3 in cardiac tissue and kidney and liver mitochondria.

### 3.6. High Dose of PQ Upregulated Pro-Apoptotic Genes in Association with Mitochondrial Apoptosis 

**Figure 6:**
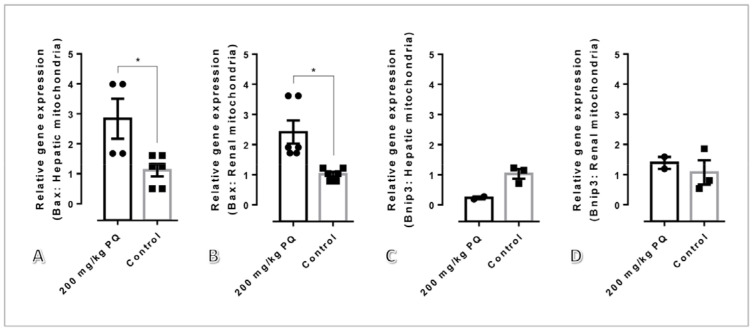
mRNA expression of pro-apoptotic related genes in renal and liver mitochondria after treated with or without 200 mg/kg of PQ; mRNA level of *Bax* (**A**,**B**) and *Bnip3* (**C**,**D**) in the hepatic (**A**,**C**) and renal (**B**,**D**) mitochondria, respectively. For subsequent figures, statistical significance of comparisons is *, *p* < 0.05.

The relative mRNA expression of mitochondrial pre-apoptotic genes in rats with and without 200 mg/kg PQ was compared (Figure 6). In mitochondria treated with 200 mg/kg of PQ, the mRNA expression of *Bax* was significantly elevated compared to the non-treated group. However, *Bnip3* gene expression was identical in both groups.

## 4. Discussion

Concerning our study, we aimed to describe the toxicopathological effects of PQ using a rat model, with an emphasis on histopathological changes in the affected tissues and mitochondrial ultrastructure in main excretory organs, particularly the liver and kidney. Subsequently, apoptotic, dynamic, antioxidative, energetic, and homeostatic mitochondrial functions were also investigated. In this study, the LD_50_ for PQ was determined to be 200 mg/kg. Hematological and blood clinical chemistry studies revealed that a high dose of PQ-induced liver and kidney damage, as evidenced by the elevation of SGPT, BUN, and creatinine (Table 2). A low number of erythrocytic indexes, particularly RBC, HGB, HCT, MCHC, and RDW, were evident in PQ-treated rats, indicating that a high dose of PQ (200 mg/kg) activated the anemic stage. In accordance with blood clinical chemistry, histopathological examination of rats treated with high doses of PQ (200 and 250 kg) revealed the presence of proximal tubular degeneration with basophilic droplet deposition (Figure 1). Rats treated with high doses of PQ (200 and 250 mg/kg) were unexpectedly found to have ventricular cardiomyopathy. In addition, a high dose of PQ (200 mg/kg) destroyed mitochondrial architecture in the liver and kidney, which was correlated with the upregulation of apoptotic markers Drp-1 and caspase-3 (Figure 2, Figure 3 and Figure 4). Similarly, expressions of hepatic and renal mitochondria, Drp-1, and caspase-3 in ventricular tissue were elevated in relation to the severity of ventricular cardiomyopathy (Figure 5). Moreover, mitochondrial apoptotic genes (*Bax*) were highly expressed in PQ-treated rats relative to non-treated rats (Figure 6). Our findings elucidated the toxic mechanism of high doses of PQ (200 mg/kg) on mitochondria and affected organs, which may serve as a safety criterion for PQ in the treatment of malaria.

Different mechanisms and functions have been described for mitochondria-mediated apoptosis or cell death in relation to the toxic effects of certain chemicals or pharmaceuticals. Among them is the utility of drug toxicity to enhance apoptotic properties on target cells or microorganisms, especially cancer cells or parasites. Shikonin, a natural naphthoquinone, promotes chemotherapeutic effect in the treatment of gastric cancer via ROS, which triggers cytochrome C for caspase cascade response and mediates the nuclear translocation of apoptosis-inducing factor [16]. The antimalarial drug mefloquine induces apoptosis in the plasmodium by stimulating the production of metacaspase and ROS [17]. In contrast, toxic properties, particularly doses of mitochondrial-mediated programmed cell death, affect not only cancer cells or parasitic cells but also normal host cells. It has been reported that chemotherapeutic drugs can cause collateral damage to normal cells, such as the occurrence of chemobrain or CICI (chemotherapy-induced cognitive impairment) due to mitochondrial dysfunction and oxidative stress [18]. Therefore, the risk/benefit ratio of the administration of drugs, chemicals, and pharmaceuticals should be considered and balanced.

Hemolytic anemia and methemoglobinemia are the most significant risks for patients with G6PD or NADH-methemoglobin reductase deficiencies, respectively, regarding the major toxic effects of PQ. Consistent with this underlining risk, the present study revealed that a high dose of PQ caused hemolytic anemia (Table 2). Currently, toxicological studies utilizing an in vivo model are being conducted to clarify the role of PQ-induced toxicity. Giovanella et al. determined, using a biochemical approach, that PQ damages DNA in the liver, brain, and kidney due to its oxidative effect, as indicated by Thiobarbituric acid reactive species (TBARS) and protein carbonyl [19]. Katewa et al. demonstrated a combination toxic effect of PQ, chloroquine, and quinine on the mitochondrial energy transduction function of rat liver mitochondria, characterized by a decrease in mitochondrial respiration rate, an uncoupling effect of mitochondrial phosphorylation, and a decrease in DNP-stimulated ATPase activity [20]. However, the effects of PQ on the kidney, liver, and heart with respect to their histopathological alterations, mitochondrial ultrastructural alterations, and mitochondria-mediated apoptosis or cell death are limited.

Mitochondria play crucial roles in both mammalian and parasitic cell apoptosis activation. Initial mitochondrial dysfunction or apoptosis consequence responses involve the release of several mitochondrial components and products via specific regulator pores on the mitochondrial membrane [21,22]. In this mechanism, Apoptotic protease activating factor-1 (Apaf-1) is an autoinhibited form. BH-3-only proteins, a regulator for translational modification, bind to antiapoptotic Bcl-2 proteins during apoptosis to activate pro-apoptotic Bax/Bak proteins, thereby promoting oligomerization and triggering Cyt-c release. Therefore, Cyt-c binds to Apaf-1 and promotes the formation of apoptosomes, resulting in apoptosis via caspase-9 and caspase-3 or -7 activation. In addition, mitochondrion architecture and apoptosis are regulated by mitochondrial dynamics and homeostasis via fission and fusion properties. Drp-1 is required for fission, a key event in mitochondrial damage and apoptosis, while OPA-1 maintains normal mitochondrial activities. Death sensitivity is significantly correlated with the interaction between Drp-1 and caspase-3 [23] or Bax [24]. Our research is the first to use an in vivo model to demonstrate that a high dose of PQ damages the renal tubule, cardiac muscle, and mitochondria in the liver and kidney via a caspase-3-mediated Drp-1 pathway. In addition to Drp-1, OPA-1, and caspase-3 expressions in the renal and hepatic mitochondria, an immunogold labeling study revealed the downregulation of MAPK in the liver mitochondria of PQ-treated rats but not in the kidney mitochondria. Consequently, MAPK is essential for certain cellular processes, including proliferation, homeostasis, apoptosis, and stress response. MAPK-Drp-1 signaling contributes to mitochondrial dysfunction and cell death [25]. However, this pathway was not observed in the present study regarding PQ-induced toxicity.

Subsequently, using a gold-labeled technique, the expression of OPA-1, HDHD-3, and Nrf-2 in renal and hepatic mitochondria was evaluated in this study. In a mouse model, the increase of OPA-1, a master regulator of mitochondrial cristae morphology, reduces mitochondrial damage from various causes, including mitochondrial DNA depletion-induced glomerulosclerosis [26] and sorafenib-induced (an anticancer agent) hepatocyte apoptosis [27]. In liver mitochondria treated with a high dose of PQ, our research revealed a downregulation of OPA-1, whereas renal mitochondria tended to exhibit a slight upregulation. A higher reserve function may be typically observed in the kidney than in the liver. In addition, Nrf-2, an antioxidative transcription factor, can reduce the cytopathic effect of oxidative stress in the renal tubule and interstitial cells [28]. Consistent with the increase in renal mitochondrial OPA-1, the upregulation of Nrf-2 in renal mitochondria exposed to a high dose of PQ would also be considered a compensatory mechanism in the kidney. Nonetheless, the expression of HDHD-3, a mitochondrial power conservation protein [9,12,13], indicated that the energetic maintenance of renal mitochondria was lower than that of liver mitochondria.

In rats administered a high dose of PQ, generalized ventricular cardiomyopathy and proximal tubular degeneration with basophilic droplet deposition predominated. In addition to mitochondrial dysregulation, cardiotoxicity and nephrotoxicity were also prominent features of PQ-induced toxicity in the present study. Myofiber degeneration and necrosis with varying degrees of inflammation, interstitial cell proliferation, and fibrosis are typical cardiomyopathy lesions. The disruption of mitochondrial oxidative phosphorylation, cell and organelle alterations, an imbalance in ion homeostasis, the interruption of essential biological molecules, the formation of toxic metabolites or free radicals, and intracellular ion level [29] changes are proposed mechanisms for substances that induce direct heart toxicity. In addition, degeneration of the proximal tubule is a non-specific lesion resulting from some causes that disturb cellular functions, such as hypoxia, disruption of ATP production, mitochondrial damage, free radical formation, peroxidation, and cell-signaling disruption. It is characterized by cell swelling with or without cytoplasmic vacuolation, pale-staining, fragmented cytoplasm, and tissue debris deposition [30]. The current study revealed histopathological changes and toxic effects of PQ at high doses on the heart and kidney. Antimalarial drugs inducing toxic effects on the heart and kidney have been reported for a decade, albeit with limited information on dose and duration. Long-term use or high doses of chloroquine induce cardiac arrhythmia by inhibiting voltage-dependent Na^+^ and Ca^2+^ channels, pacemaker channels, and voltage-dependent K^+^ channels [31]. Moreover, acute megaloblastic anemia, exfoliative dermatitis, and symptomatic pancytopenia are complications end-stage renal failure. In patients allergic to quinine, neutropenia, anemia, thrombocytopenia, and disseminated intravascular coagulopathy [32] can accompany acute renal failure. Furthermore, artesunate decreases glomerular infiltration rate, boosts renal blood flow, and accelerates Na, Cl, and K excretion [33].

## 5. Conclusions

In summary, the LD_50_ for PQ was 200 mg/kg. In addition to hematotoxicity as demonstrated by hemolytic anemia and hepatotoxicity as demonstrated by the marked increase in the liver enzyme (SGPT), a high dose of PQ caused ventricular cardiomyopathy and proximal tubular degeneration as well as mitochondrial dysregulation in the kidney and liver concerning Drp-1-mediated caspase-3 cell death. In addition, a high dose of PQ increased the upregulation of the apoptotic genes *Bax*. These underlying mechanisms identified in the present study may be useful for the preventive use of PQ in the treatment of malaria and other diseases as well as for a better understanding of the toxicopathological effects of PQ.

## Figures and Tables

**Table 1:** Apoptotic gene primers used for qRT-PCR.

Gene	Primers	
*18S*	F	5′GCCGCTAGAGGTGAAATTCTTG3′
	R	5′GAAAACATTCTTGGCAAATGCTT3′
*Bax*	F	5′AGAACCATCATGGGCTGGAC3′
	R	5′AGATGGTCACTGTCTGCCATGT3′
*Bnip3*	F	5′CAGAGCGGGGAGGAGAAC3′
	R	5′GAAGCTGGAACGCTGCTC3′

**Table 2:** Complete blood count and blood clinical chemistry comparing between rats treated with or without PQ; Mean ± SD.

Parameters	Normal Range ^#^	Control	200 mg/kg of PQ
WBC (10^6^/µL)	3.51–4.95	4.92 ± 7.37	5.07 ± 5.09 ^↑^
RBC (10^6^/µL)	8.64–9.90	9.01 ± 0.27	7.56 ± 0.77 * ^↓^
HGB (g/dL)	16.76–18.80	17.15 ± 0.07	15.65 ± 1.62 ^↓^
HCT (%)	52.65–60.25	54.55 ± 0.49	47.45 ± 5.02 * ^↓^
MCV (fl)	59.18–62.68	63.55 ± 3.18	62.75 ± 0.21
MCH (pg)	18.69–20.71	20.20 ± 0.84	20.70 ± 0.00
MCHC (g/dL)	31.12–31.88	31.75 ± 0.02	22.95 ± 0.07 * ^↓^
PLT (10^3^/µL)	667.62–941.38	1032.50 ± 132.22 ^↑^	941.50 ± 94.04
RDW (%)	15.85–19.73	15.00 ± 0.56	12.45 ± 0.21 * ^↓^
Neutrophils (%)	4.75–18.71	13.70 ± 10.32	21.50 ± 7.77 ^↑^
Lymphocytes (%)	72.85–86.91	80.20 ± 4.52	74.00 ± 11.31
Eosinophils (%)	0.84–1.50	0.05 ± 0.07	0.50 ± 0.70
Basophils (%)	0.00–0.73	0.35 ± 0.49	0.00 ± 0.00
Monocytes (%)	5.38–8.36	5.70 ± 5.23	4.00 ± 4.24
Blood urea nitrogen (mg/dL)	18.22–23.24	21.10 ± 0.98	28.10 ± 16.70 ^↑^
Creatinine (mg/dL)	0.63–0.71	0.16 ± 0.01	0.63 ± 0.00 *
SGPT (U/L)	43.03–63.33	36.84 ± 6.84	593.60 ± 124.87 ** ^↑^
SGOT (U/L)	81.77–105.69	30.55 ± 2.05	55.3 ± 10.32

WBC, white blood cell; RBC, red blood cell; HCT, hematocrit; MCV, mean corpuscular volume; MCH, mean corpuscular hemoglobin; MCHC, mean corpuscular hemoglobin concentration; PLT, platelet; RDW, red cell distribution width; SGPT, serum pyruvate-glutamate transaminase; SGOT, serum glutamate-oxaloacetate transaminase; #, in-house value from animal supplier. * *p*-value < 0.05 or ** *p*-value < 0.01; significant difference between treatment and non-treatment groups, ^↑^ or ^↓^; increase or decrease when comparing with normal ranges, respectively.

## Data Availability

Not applicable.
